# Effect of a prevention bundle for internal jugular vein malposition of peripherally inserted central catheters in children: a single-center before–after study

**DOI:** 10.3389/fped.2026.1939170

**Published:** 2026-09-03

**Authors:** Yusuke Tokuda, Kentaro Ide, Hajime Okuwaki, Hidehito Ota, Shotaro Matsumoto

**Affiliations:** 1 Critical Care Medicine, National Center for Child Health and Development, Tokyo, Japan; 2 Department of Pediatrics, The University of Tokyo Hospital, Tokyo, Japan; 3 Department of Pediatrics, University of Tsukuba Hospital, Ibaraki, Japan

**Keywords:** catheter malposition, internal jugular vein, pediatric intensive care unit, pediatrics, peripherally inserted central catheter

## Abstract

**Background:**

Peripherally inserted central catheters (PICCs) are widely used in pediatric intensive care units, but bedside insertion without fluoroscopy often results in catheter tip malposition into the internal jugular vein (IJV). We implemented a three-component prevention bundle consisting of head rotation toward the insertion side, arm abduction, and neck ultrasonography confirmation of the guidewire, and evaluated its effect on IJV catheter malposition.

**Methods:**

This before–after study was performed on patients aged <16 years who underwent PICC placement during the pre- and post-bundle periods. The primary analysis compared IJV catheter malposition between the pre-bundle and fully adherent post-bundle groups. Secondary analysis evaluated adherence to individual bundle components and their association with IJV catheter malposition.

**Results:**

We compared 168 pre-bundle patients with 112 fully adherent post-bundle patients. The IJV catheter malposition rate decreased from 11.9% (20/168) to 6.3% (7/112). However, this reduction was not statistically significant (*p* = 0.15). In unadjusted analysis, full bundle adherence was associated with lower odds of malposition compared with the pre-bundle group (OR, 0.49; 95% CI, 0.20–1.21; *p* = 0.12). After adjustment for age and primary diagnostic category, the odds ratio decreased to 0.41 (95% CI, 0.16–1.02; *p* = 0.06). In multivariable analysis of the individual bundle components, head rotation was associated with lower odds of malposition (adjusted OR, 0.22; 95% CI, 0.06–0.76; *p* = 0.02), whereas arm abduction and neck ultrasonography were not.

**Conclusions:**

The effect of bundle implementation was limited. A simpler, more reliable bundle should be investigated.

## Introduction

Peripherally inserted central catheters (PICCs) are commonly used in the pediatric intensive care unit (PICU) to secure long-term venous access and administer hyperosmolar medications. In critically ill children with respiratory failure or hemodynamic instability, PICCs are often placed at the bedside without fluoroscopy. Under non-fluoroscopic conditions, catheter tip malposition is common, and 4%–13% of patients experience malposition into the internal jugular vein (IJV catheter malposition) (1–4). Tip malposition is associated with thrombosis, phlebitis, impaired infusion or blood withdrawal, and an increased risk of infection due to catheter reinsertion (5, 6). Thus, effective strategies that can prevent malposition are clinically important. Several procedural maneuvers that can reduce the risk of IJV catheter malposition during bedside PICC placement have been proposed. Head rotation toward the insertion side can narrow the angle between the subclavian vein and the IJV and can partially compress the IJV (7–9). Arm abduction has been traditionally recommended to straighten the venous pathway toward the superior vena cava and facilitate caudal progression of the guidewire (6). In addition, neck ultrasonography confirmation of the guidewire position allows real-time identification of inadvertent IJV entry and enables immediate correction before catheter advancement (10, 11).

However, in pediatric PICC placement, there are no clear data on which preventive measures are most effective and feasible in routine bedside practice. Therefore, a three-component prevention bundle that comprises head rotation toward the insertion side, arm abduction, and neck ultrasonography confirmation was implemented in 2023. These three interventions were selected because they are simple to perform at the bedside and do not require additional equipment or specialized costs in the PICU setting. The combined effect of these interventions in pediatric PICC placement has not been systematically evaluated. The current study primarily aimed to evaluate the effect of this prevention bundle by comparing the rates of IJV catheter malposition before and after its introduction. The secondary aim was to examine the individual effectiveness of each bundle component during the post-bundle period.

## Methods

A single-center retrospective before–after study was conducted at the PICU of a tertiary children's hospital in Japan. The pre-bundle period was defined as the time from May 2017 to September 2018, which is before the implementation of the prevention bundle. The post-bundle period was defined as the time from January 2023 to December 2023, which is after bundle implementation and dissemination.

All patients who underwent PICC insertion in the PICU during the study periods were screened for eligibility. Patients aged ≥16 years, those with venous anatomical abnormalities (e.g., congenital anomalies and postsurgical alterations of the access veins), those who did not complete the PICC procedure, those with PICC tip malposition into non-IJV vessels, and those with missing essential data regarding bundle adherence were excluded from the study.

PICC insertions were typically performed by pediatric intensivists with at least 5 years of clinical experience. Before performing PICC procedures independently, all operators completed standardized training, which included a review of PICC indications and complications, and a simulation-based assessment of insertion techniques. The PICCs used in this study included 3 Fr single-lumen, 4 Fr single-lumen, 18 G double-lumen, and 4.5 Fr double-lumen catheters. All PICCs were inserted using the modified Seldinger technique with guidewire assistance. The basilic or brachial vein in the upper arm was generally selected as the access site and cannulated under real-time ultrasonography guidance. Catheter tip position was routinely confirmed on post-procedural chest radiography.

The bundle comprised three components: (1) head rotation—during guidewire advancement, the patient's face was rotated toward the insertion side; (2) arm abduction—during guidewire advancement, the punctured upper arm was abducted to an angle of ≥90°; and (3) neck ultrasonography confirmation—after guidewire advancement and before PICC catheter advancement, a high-frequency hockey-stick probe was used to assess the presence of the guidewire in the IJV. Neck ultrasonography was performed by a PICU staff physician other than the catheter inserter, all of whom had completed institutional training in vascular ultrasonography and had at least 9 years of postgraduate clinical experience. This institutional training included ultrasonographic guidewire localization during vascular access procedures but was not specifically designed for detecting guidewire malposition in the internal jugular vein. If the guidewire was visualized within the IJV, it was withdrawn and repositioned until it was no longer found in the IJV. Bundle adherence for each component was recorded as yes or no by the operator immediately after the procedure.

The primary outcome was the rate of IJV catheter malposition on post-procedural chest radiography. IJV catheter malposition was defined as the placement of the catheter tip within the IJV on post-procedural chest radiography. All radiograph images were routinely interpreted by pediatric radiologists as part of standard clinical care, and the documented radiology reports were retrospectively reviewed for this study. For the primary analysis, the rates of IJV catheter malposition were compared between the pre-bundle group and the completely adherent post-bundle group. Because a significant baseline age difference was identified between the pre-bundle and completely adherent post-bundle groups, an additional *post hoc* logistic regression analysis was performed. The model included study group, age, and primary diagnostic category as independent variables. As an additional sensitivity analysis, all patients in the post-bundle period were compared with the pre-bundle group regardless of bundle adherence to evaluate the overall effect of bundle implementation. For the secondary analysis, adherence to each bundle component during the post-bundle period was assessed, and the associations between each bundle component and IJV catheter malposition were evaluated using univariable and multivariable logistic regression analyses.

Categorical variables were presented as numbers (percentages), and continuous variables were expressed as medians with interquartile ranges (IQRs). Categorical variables were compared using the Fisher's exact test, and continuous variables were assessed using the Mann–Whitney U test. To evaluate the independent association of each bundle component with IJV catheter malposition, both univariable and multivariable logistic regression analyses were performed. The multivariable model simultaneously included the three bundle components (head rotation, arm abduction, and neck ultrasonography confirmation) as covariates. Two-sided *p* values of <0.05 indicated statistically significant differences. All analyses were performed using R version 4.3.1 (R Foundation for Statistical Computing, Vienna, Austria).

The sample size was estimated assuming an IJV catheter malposition rate of 11% in the pre-bundle period according to our previous report (4) and 1% in the post-bundle period based on a previous report (11), a power of 0.80, a two-sided *α* value of 0.05, and a pre- to post-bundle case ratio of 3:2. Based on these assumptions, the required sample sizes were calculated as 140 in the pre-bundle group and 94 in the post-bundle group. To account for an anticipated 20% nonadherent rate to the bundle during the post-bundle period, a total of 118 patients would be required.

The institutional review board of the National Center for Child Health and Development approved this study (approval number: 2024–031). In accordance with the national ethical guidelines for medical and health research involving human subjects, informed consent was waived and an opt-out approach was used, thereby allowing patients and guardians to decline participation. The current study was reported in accordance with the Strengthening the Reporting of Observational Studies in Epidemiology statement for observational cohort studies.

## Results

**Figure 1:**
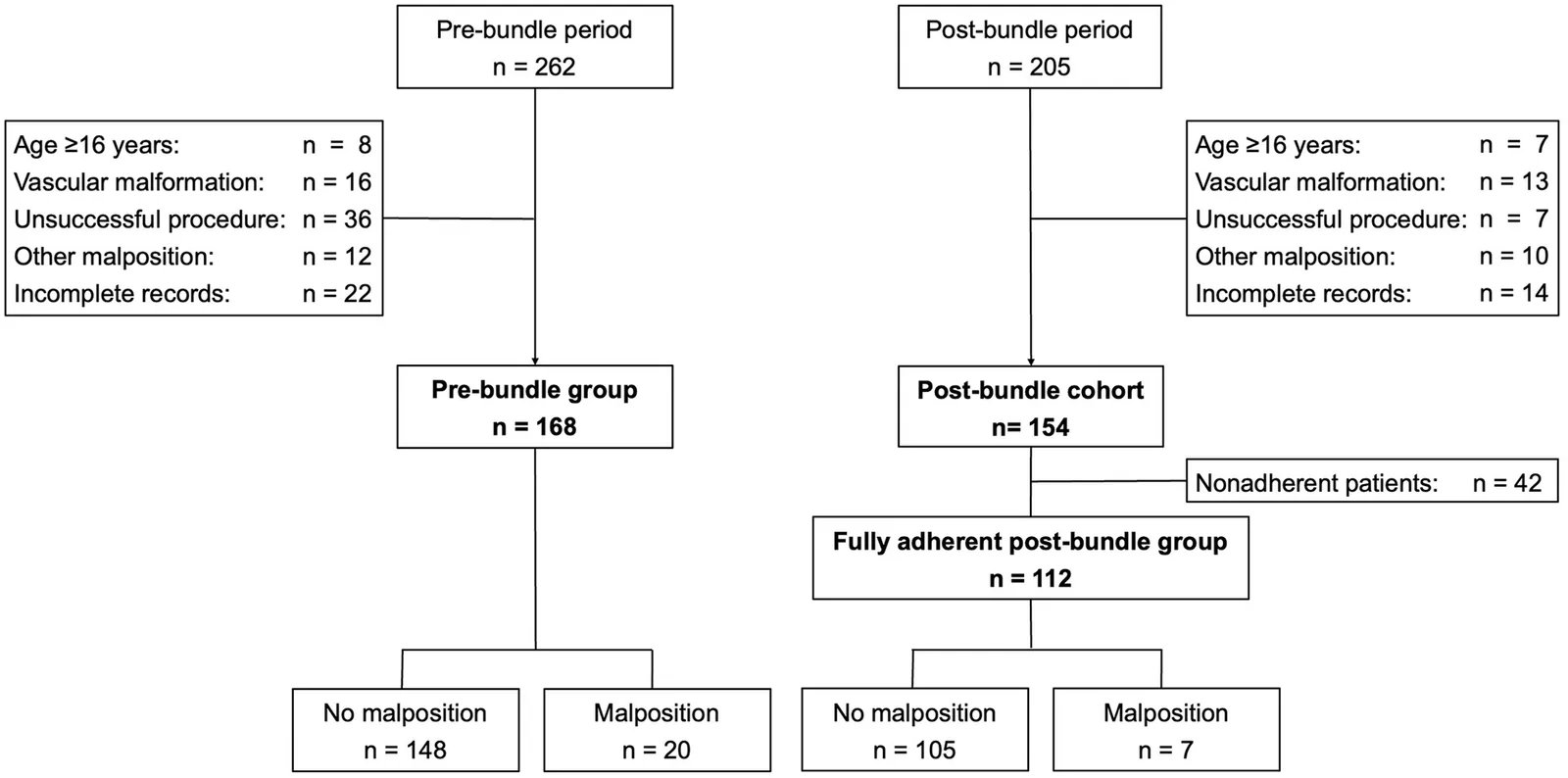
Flow diagram of the study. Flow of patient inclusion in the pre-bundle and post-bundle periods and classification of post-bundle patients according to complete versus partial or nonadherence to the three-component prevention bundle.

During the study periods, 168 patients in the pre-bundle group were included in the primary analysis. In the post-bundle period, 154 patients were assessed. Among them, 112 who completely adhered to all three bundle components were included in the primary analysis. Meanwhile, the remaining 42 patients were classified under the non-adherent group (Figure 1). Table 1 shows the baseline characteristics of the pre-bundle and completely adherent post-bundle groups. The pre-bundle group had a lower median age (months) than the completely adherent post-bundle group [16 (7–49) vs. 35 (10–73) months, *p* = 0.001]. The distribution of primary diagnostic categories upon PICU admission and the insertion side did not differ significantly between the two groups. The rate of IJV catheter malposition decreased from 11.9% (20/168) in the pre-bundle group to 6.3% (7/112) in the completely adherent post-bundle group. However, this difference did not reach statistical significance (*p* = 0.15). Because the post-bundle group was significantly older than the pre-bundle group, and patient case-mix may also have differed between the groups, an additional *post hoc* logistic regression analysis was performed adjusting for age and primary diagnostic category. In the unadjusted logistic regression analysis, the odds ratio (OR) for IJV catheter malposition comparing the completely adherent post-bundle group with the pre-bundle group was 0.49 [95% confidence interval (CI), 0.20–1.21; *p* = 0.12]. After adjustment for age and primary diagnostic category, the association became stronger but remained statistically non-significant (adjusted OR, 0.41; 95% CI, 0.16–1.02; *p* = 0.06).

**Table 1:** Baseline characteristics of the pre-bundle and completely adherent post-bundle groups.

Number of patients, *n*	Pre-bundle group *n* = 168	Completely adherent post-bundle group *n* = 112	*p*-value
Male sex, *n* (%)	96 (57.1)	61 (54.5)	0.71
Age, months, median (IQR)	16 (7–49)	35 (10–73)	0.001
Height, cm, median (IQR)	73 (61–95)	86 (66–114)	0.003
Body weight, kg, median (IQR)	9.5 (6.4–13.8)	10.9 (7.0–18.2)	0.03
Primary diagnostic category upon PICU admission, *n* (%)			0.36
Hepatic disease	74 (44.0)	45 (40.2)	
Central nervous system disease	36 (21.4)	35 (31.2)	
Cardiac disease	25 (14.9)	13 (11.6)	
Respiratory disease	13 (7.7)	5 (4.5)	
Others	20 (11.9)	14 (12.5)	
Invasive mechanical ventilation, *n* (%)	72 (42.9)	39 (34.8)	0.21
Right upper extremity insertion, *n* (%)	85 (50.6)	56 (50.0)	1.00

To assess the overall effect of bundle implementation, an additional sensitivity analysis was performed in which all 154 patients in the post-bundle period were compared with the pre-bundle group regardless of bundle adherence. The IJV catheter malposition rate was 11.0% (17/154) in the post-bundle period and 11.9% (20/168) in the pre-bundle group, with no significant difference between the groups (*p* = 0.86).

During the post-bundle period, 42 patients did not adhere to at least one component. The baseline characteristics of the completely adherent and non-adherent groups were generally comparable (Table 2), although invasive mechanical ventilation at the time of PICC insertion was more frequent in the non-adherent group (57.1% vs. 34.8%, *p* = 0.02). The IJV catheter malposition rates were 6.3% (7/112) in the completely adherent group and 23.8% (10/42) in the non-adherent group (*p* = 0.004). The adherence rates for the individual bundle components were 89.0% (137/154) for head rotation, 92.2% (142/154) for arm abduction, and 80.5% (124/154) for neck ultrasonography confirmation. Among the 124 patients who underwent neck ultrasonography, 15 (12.1%) patients experienced guidewire malposition into the IJV, and the issue was successfully corrected in 14 (93.3%) patients. In contrast, although guidewire malposition was not detected on neck ultrasonography, nine (7.3%) patients had IJV catheter malposition.

**Table 2:** Characteristics of the patients and bundle adherence in the post-bundle cohort.

Number of patients, *n*	Completely adherent post-bundle group *n* = 112	Nonadherent group *n* = 42	*p*-value
Male sex, *n* (%)	61 (54.5)	19 (45.2)	0.37
Age, months, median (IQR)	35 (10–73)	39 (8–70)	0.64
Height, cm, median (IQR)	86 (66–114)	82 (64–105)	0.45
Body weight, kg, median (IQR)	10.9 (7.0–18.2)	10.5 (6.6–18.5)	0.63
Primary diagnostic category upon PICU admission, *n* (%)			0.09
Hepatic disease	45 (40.2)	11 (26.2)	
Central nervous system disease	35 (31.2)	10 (23.8)	
Cardiac disease	13 (11.6)	6 (14.3)	
Respiratory disease	5 (4.5)	6 (14.3)	
Others	14 (12.5)	9 (21.4)	
Invasive mechanical ventilation, *n* (%)	39 (34.8)	24 (57.1)	0.02
Right upper extremity insertion	56 (50.0)	22 (52.4)	0.86
Nonadherence to each bundle component, *n* (%)			
Head rotation	0 (0)	17 (40.5)	
Arm abduction	0 (0)	12 (28.6)	
Neck ultrasonography confirmation	0 (0)	30 (71.4)	

Table 3 presents the results of the univariable and multivariable logistic regression analyses of the bundle components. In the univariable analysis, head rotation (OR, 0.16; 95% CI, 0.05–0.52; *p* = 0.002) and neck ultrasonography confirmation (OR, 0.29; 95% CI, 0.10–0.84; *p* = 0.02) were associated with lower odds of IJV catheter malposition, whereas arm abduction was not (OR, 0.33; 95% CI, 0.08–1.36; *p* = 0.12). After adjustment for the other bundle components, only head rotation remained independently associated with lower odds of IJV catheter malposition (adjusted OR, 0.22; 95% CI, 0.06–0.76; *p* = 0.02). Neither arm abduction (adjusted OR, 0.59; 95% CI, 0.13–2.67; *p* = 0.49) nor neck ultrasonography confirmation (adjusted OR, 0.42; 95% CI, 0.14–1.33; *p* = 0.14) was independently associated with IJV catheter malposition.

**Table 3:** Associations between adherence to individual bundle components and IJV catheter malposition.

Bundle component (adherence rate)	Unadjusted odds ratio (95% CI)	*p-*value	Adjusted odds ratio (95% CI)	*p-*value
Head rotation (137/154)	0.16 (0.05–0.52)	0.002	0.22 (0.06–0.76)	0.02
Arm abduction (142/154)	0.33 (0.08–1.36)	0.12	0.59 (0.13–2.67)	0.49
Neck ultrasonography confirmation (124/154)	0.29 (0.10–0.84)	0.02	0.42 (0.14–1.33)	0.14

## Discussion

In this single-center before–after study, complete adherence to the three-component bundle for preventing IJV catheter malposition was associated with a lower IJV catheter malposition rate (11.9% vs. 6.3%). This reduction did not reach statistical significance (*p* = 0.15), although the study may have been underpowered to detect the observed effect size. Only head rotation remained independently associated with lower odds of IJV catheter malposition in the multivariable analysis, whereas no independent association was observed for the other bundle components.

The primary analysis did not show a statistically significant reduction in IJV catheter malposition despite a numerically lower rate. This finding may be explained by the observed post-bundle rate of IJV catheter malposition being higher than anticipated. Our sample size calculation assumed a post-bundle IJV catheter malposition rate of approximately 1% based on a previous adult study (11). Meanwhile, the observed rate in the completely adherent group was 6.3%. Consequently, the study may have been underpowered to detect the more modest effect size observed in this pediatric cohort. Therefore, the absence of statistical significance should be interpreted cautiously, as a type II error cannot be excluded. This discrepancy may reflect differences between adult and pediatric populations, including pediatric-specific procedural challenges. In pediatric patients, adequate sedation is often required during PICC placement. However, maneuvers such as head rotation, arm abduction, and repeated neck ultrasonography examination may provoke patient movement or arousal. As a result, these interventions may be applied incompletely or inconsistently in clinical practice. In addition, ultrasonography confirmation of guidewire position in the pediatric neck is technically challenging due to the limited field of view and restricted acoustic windows created by devices such as central venous catheters and tracheostomy tubes. Taken together, these pediatric-specific procedural challenges may have attenuated the overall efficacy of the prevention bundle.

In the analysis of individual bundle components, head rotation toward the insertion side was independently associated with a lower risk of IJV catheter malposition, which is consistent with a previous report (7). In contrast, arm abduction did not show a clear independent association with malposition reduction. Although arm abduction is performed empirically, prior studies have not shown that the use of a single maneuver has evident benefit (6, 10), and the present study showed similar findings. Neck ultrasonography confirmation was also not independently associated with lower odds of IJV catheter malposition in the multivariable analysis, which may be partly explained by false-negative ultrasonography assessment results. In particular, 7.3% of patients had IJV catheter malposition on post-procedural radiography even though the guidewire was not visualized within the IJV on ultrasonography. These findings indicate that ultrasonography confirmation alone may be insufficient and that dedicated training focused on identifying guidewire malposition in the internal jugular vein during PICC insertion may further improve the diagnostic performance of neck ultrasonography and reduce false-negative findings.

Based on our findings, future efforts for preventing IJV catheter malposition during pediatric bedside PICC placement should focus on head rotation toward the insertion side, along with ultrasonography-based confirmation strategies refined for pediatric practice. In addition, future studies should evaluate whether combining these simple maneuvers with adjunct approaches, such as ultrasonography-guided manual compression of the IJV during catheter advancement (10) and advanced tip confirmation technologies, including intracavitary electrocardiogram or magnetic tracking-based tip confirmation systems [e.g., Sherlock 3CG® (12–14)], can further reduce malposition rates. Prospective studies must be carried out to assess the feasibility, efficacy, and generalizability of these integrated strategies in pediatric clinical practice.

This study has several limitations. First, it was a single-center retrospective before–after study, and differences in patient case-mix, operators, or practice over time might have introduced temporal confounding. Second, in the pre-bundle period, preventive measures were left to individual operators, and some patients might have received partial prevention strategies. Third, the sample size calculation was based on an expected reduction in IJV catheter malposition from 11% to 1%, which was derived from a previous adult study. As the observed reduction in the present pediatric cohort was more modest than expected (11.9% to 6.3%), the study may have been underpowered to detect this effect. Therefore, the lack of statistical significance should be interpreted with caution because a type II error cannot be excluded. Based on the observed effect size, larger multicenter studies with substantially larger sample sizes are needed to confirm the effectiveness of the prevention bundle.

## Conclusion

In conclusion, complete adherence to the three-component bundle did not significantly reduce the rate of IJV catheter malposition in pediatric bedside PICC placement. Among the bundle components, head rotation toward the insertion side was independently associated with a lower risk of malposition. Meanwhile, the other components had more limited effects. These findings suggest that a streamlined bundle with greater emphasis on procedural accuracy warrants prospective evaluation in pediatric bedside PICC placement. In addition, because a negative neck ultrasonography examination did not reliably exclude subsequent IJV catheter malposition, post-procedural catheter tip confirmation remains necessary.

## Data Availability

The raw data supporting the conclusions of this article will be made available by the authors, without undue reservation.
